# Splenectomy and proximal lieno-renal shunt in a factor five deficient patient with extra-hepatic portal vein obstruction

**DOI:** 10.1186/1471-2482-6-7

**Published:** 2006-05-19

**Authors:** Srinivas Prabhu Chava, Sujoy Pal, Supriyo Ghatak, Rajat Kumar, Peush Sahni, Tushar Kanti Chattopadhyay

**Affiliations:** 1 Department of G.I. Surgery, All India Institute of Medical Sciences, New Delhi, 110029, India; 2 Department of Hematology, All India Institute of Medical Sciences, New Delhi, 110029, India

## Abstract

**Background:**

The clinico-surgical implication and successful management of a rare case of factor five (V) deficiency with portal hypertension and hypersplenism due to idiopathic extra-hepatic portal venous obstruction is presented.

**Case presentation:**

A 16-year old boy had gastro-esophageal variceal bleeding, splenomegaly and hypersplenism. During preoperative workup prolonged prothrombin time and activated partial thromboplastin time were detected, which on further evaluation turned out to be due to factor V deficiency. Proximal lieno-renal shunt and splenectomy were successfully performed with transfusion of fresh frozen plasma during and after the surgical procedure. At surgery there was no excessive bleeding. The perioperative course was uneventful and the patient is doing well on follow up.

**Conclusion:**

Surgical portal decompressive procedures can be safely undertaken in clotting factor deficient patients with portal hypertension if meticulous surgical hemostasis is achieved at operation and the deficient factor is adequately replaced in the perioperative period.

## Background

Congenital deficiency of factor V (parahemophilia) is a rare inherited disorder of coagulation which appears to follow an autosomal recessive or partially dominant pattern [1-4]. Although the clinical features are rather mild compared with hemophilia, any surgical procedure undertaken without replacement therapy is uniformly complicated by excessive hemorrhage [1,2,5]. As factor V concentrate is not available, fresh frozen plasma (FFP) is the only available source of factor V at the present time. We report a boy with extra-hepatic portal venous obstruction (EHPVO), portal hypertension and upper gastrointestinal (UGI) hemorrhage who also had factor V deficiency. Although many congenital anomalies such as cleft palate, mental retardation, short stature, syndactyly, atrial septal defect, ventricular septal defect, coarctation of aorta, duplication of the genitourinary collecting system have been associated with congenital deficiency of factor V [1-4], the simultaneous occurrence of factor V deficiency with EHPVO has not been described till date. EHPVO is a relatively common cause of portal hypertension in India, especially in the younger age group [6,7]. Many causes, such as congenital, umbilical sepsis, recurrent childhood diarrhoea, dehydration, intra-abdominal infection, malnutrition and neonatal exchange transfusion have been postulated [6,8]. The aetiology of EHPVO still remains unclear and in most it is labelled idiopathic.

## Case presentation

A 16-year old boy was admitted to the Department of Gastrointestinal Surgery, All India Institute of Medical Sciences (AIIMS), for the management of recurrent episodes of UGI bleeding. He had the first episode of hematemesis and melena at the age of 11 years. He was managed elsewhere with 3 units of blood transfusion and endoscopic sclerotherapy for esophageal varices. He had his esophageal varices obliterated with 10 sessions of endoscopic sclerotherapy. He had an episode of recurrent UGI bleeding 9 months before presentation that was associated with hemodynamic instability, for which he received 4 units of blood transfusion and was subsequently referred to our centre. Additionally, he complained of a progressively growing lump in the left upper abdomen since the age of 3 years. He never had jaundice, ascites or encephalopathy. There was no history of any gum bleed, epistaxis, ecchymosis or prolonged bleeding from minor cuts. His general physical examination was normal; abdominal examination revealed a 6 cm non-tender smooth splenomegaly and a normal liver span. UGI endoscopy at AIIMS revealed 3 columns of grade II esophageal varices and gastric fundal varices. Duplex ultrasound scan of the abdomen showed a normal liver, splenomegaly and obliterated portal vein with multiple collaterals (portal cavernoma) in the hilum of the liver. Splenic vein calibre was 12 mm. Although his hemoglobin was normal (13 gm/dl) the hemogram revealed features of hypersplenism (low total leukocyte count: 2300/mm^3 ^and low platelets: 70,000/mm^3^). Liver function tests, other than a prolonged prothrombin time (PT), were within normal limits (viz., serum bilirubin: 0.7 mg/dl, serum alkaline phosphatase: 190 U/dl, serum aspartate transaminase: 32 U/dl, serum alanine transaminase: 28 U/dl). PT, activated partial thromboplastin time (APTT), and thromboelastogram (TEG) were significantly deranged (Table 1). Assay for clotting factors revealed factor V deficiency (activity less than 20% of normal). Other clotting factor concentrations were normal and the screening for factor inhibitors was negative.

The possible problems during surgery with regard to excessive bleeding were explained to the parents and an informed written consent was obtained. A clinical haematologist saw the patient and a plan for pre and peri-operative FFP infusions with coagulation monitoring was decided upon. The patient was transfused 1500 ml of FFP the day before operation. Following transfusion the PT, APTT and TEG significantly improved (Table 1), although they did not normalize. He was transfused 900 ml of FFP over 3 hours just before the start of the operation. He underwent splenectomy and proximal lieno-renal shunt (PLRS) through a left thoraco-abdominal incision (8^th ^intercostal space) using a standardized surgical technique described by us previously [7]. The intra-operative blood loss was 1000 ml. During the operation he received two units of packed cells and 750 ml of FFP. The operative time was 4 hours and the patient remained hemodynamically stable throughout the procedure. Postoperatively he received 600 ml of FFP every 12 hours for the first 48 hours followed by 450 ml of FFP every 12 hours for the next 48 hours. There was no bleeding from the drain sites and the wounds healed well without haematoma formation. Abdominal drain and chest tube were removed on 3^rd ^and 8^th ^postoperative days, respectively. Patient had high spiking fever ranging between 39°C–40°C in the initial postoperative period. Although the patient was initially started on i.v. amoxicillin with clavulinic acid and amikacin as per our protocol, antibiotics were changed to teicoplanin and piperacillin-tazobactam on the 3^rd ^postoperative day. He became afebrile on the 6^th ^postoperative day and intravenous antibiotics were continued till 10^th ^postoperative day. Cultures from blood, urine and drain fluids were repeatedly sterile. An ultrasound evaluation did not reveal any subphrenic or intra-abdominal collection. He was discharged on the 12^th ^postoperative day. Following discharge, at 4 months follow-up, the patient has had no recurrence of variceal bleeding and no evidence of hypersplenism (hemoglobin- 11 gm/dl, total leukocyte count- 6600/mm^3^, platelets- 325,000/mm3^3^). A Doppler ultrasound study showed a patent lieno-renal shunt.

## Discussion

Factor V (labile factor or proaccelerin) is a large single chain glycoprotein (2196 amino acids) and the gene for factor V is located on chromosome 1 q21-q25. The average plasma concentration of factor V is 6.6 μg/ml (20 nmol/L). Platelets contain approximately 18–25% of the factor V in whole blood, within ά granules. Liver appears to be the primary site of factor V biosynthesis. Procofactor V does not bind factor Xa and is essentially completely inactive. The primary catalyst of factor V activation in-vivo is ά-thrombin. Activation of procofactor V yields the functional form of factor Va which acts as a cofactor for the serine protease factor Xa in the prothrombinase complex and leads to a rate enhancement of almost 300,000 fold in the process of factor Xa activation of prothrombin [4]. Factor V is an extremely labile protein and is rapidly degraded in stored blood or plasma. As no factor V concentrate or recombinant factor V is available, treatment consists of fresh frozen plasma (FFP). The half-life of factor V is probably about 12 to 15 hours although a range from 4.5 to 36 hours has been described [1-5,9]. More rapid disappearance is expected during surgery due to bleeding. In this context it is pertinent to state that infusions of solvent/detergent (S/D) plasma have been shown to have significant reductions in the activity of factors V, VIII and protein S. Hence its use is not considered appropriate for patients with factor V deficiency undergoing surgery, even though the risks of viral transmission are significantly lower with S/D plasma when compared to FFP [10].

Congenital deficiency of factor five or parahemophilia is an extremely rare disorder with an estimated prevalence of 1 in 1 million [11]. Less than 200 cases of inherited factor five deficiency have been reported since its first description by Owren in 1947 in a woman with menorrhagia [12]. Combined factor V and VIII deficiency has also been described and probably occurs more commonly than factor V deficiency alone. It is an autosomal recessive trait [1,2,4], manifests clinically only in patients who inherit defective genes from both the parents. Other modes of inheritance have been implicated in certain kindreds [2,3]. In heterozygotes, the level of factor five in plasma is approximately half of the normal and the carriers are easily identifiable by routine laboratory studies [4]. A recent study detailing molecular analysis of factor V gene in patients with severe factor V deficiency revealed 9 different mutations [13]. These researchers felt that there was a high level of allelic heterogeneity in these cases with a large number of "private" mutations in FV-deficient families. For this reason they recommend a full mutational screening of FV gene in affected individuals for a complete molecular diagnosis.

The clinical features are rather mild compared with hemophilia and the patients experience few bleeding problems in daily life but experience bleeding complications following dental extraction, severe trauma or surgery. The goal of hematologic management in the perioperative period is to maintain sufficient levels of clotting factors for a sufficient period of time to prevent bleeding complications. Conventional regimen for patients with congenital clotting factor deficiency undergoing major elective surgery is to bring patient's plasma level to 100% just prior to surgery, maintain a level greater than 60% for 4 days and a level greater than 40% for 4 days [14]. However, the safe plasma factor V level for major surgery is thought to be 25% to 33% and there are case reports where elective surgery has been successfully performed above that level. In one of the reports bleeding occurred 54 and 78 hours following dental extraction and factor V levels at these times were 24% and 18% of normal, respectively [5]. Hence, it is important to maintain adequate levels of factor V in the postoperative period. Recommended dosage of FFP replenishment in these patients undergoing major surgery is 20 ml/kg of body weight as loading dose over 3–4 h to prevent the adverse effects of volume overload followed by 10 ml/kg every 12–24 hours [9], as has been used by us. This high dose regimen recommended for severe factor V deficient individuals was deliberately chosen because the exact level of factor V activity was not available to us. This technical limitation forced us to 'err' on the side of surgical safety as haemorrhage during the surgery for portal hypertension could have been lethal.

The simultaneous occurrence of factor V deficiency with EHPVO has not been described till date. We have not encountered a single case of factor V deficiency in 808 cases of EHPVO we have operated on since 1976 [[6,7] and unpublished data]. EHPVO is a relatively common cause of portal hypertension in India, especially in the younger age group [6,7]. Splenectomy and PLRS is an effective treatment (secondary prophylaxis) for this condition and is successful in preventing variceal rebleeding in almost 90% of cases [7]. This operation also takes care of the large splenomegaly and associated hypersplenism. As the liver function is normal in these patients, PLRS is not associated with post shunt encephalopathy. PLRS is considered a major undertaking with a potential for significant morbidity and mortality, and many prefer endoscopic sclerotherapy (EST) for the control of esophageal varices. These patients usually have multiple collateral vessels in the lienorenal and gastrosplenic ligaments, splenic attachments to the diaphragm and in the retroperitoneum around splenic and renal vessels. As mobilization of the spleen and splenic vein may cause significant hemorrhage, painstaking ligation of these collaterals during splenectomy and meticulous dissection of often thin walled, distended and friable splenic vein from pancreatic tail is required. However, in centers with substantial experience the complication rate is low; our own elective mortality rate for PLRS is 0.7%, variceal rebleeding rate is 11% and encephalopathy rate is zero [7]. Moreover, PLRS is a one time procedure for EHPVO which treats the painfully enlarged spleen, hypersplenism and portal hypertension all at the same time. It eliminates the risk of variceal bleeding immediately whereas EST takes an average of 8 sittings over many months to obliterate varices [15]. The patient remains at risk of rebleed until the varices are obliterated. Also, following EST the portal system is not decompressed and these patients are at 16% risk of variceal recurrence in esophagus [15] and 11% develop new varices in stomach [16], with consequent high rebleed rates, as had happened in our patient following EST. These patients are also at risk of development of ectopic varices and bile duct varices with portal biliopathy following EST. Hence there is a strong case for decompressive shunt surgery in these EHPVO patients particularly in Indian conditions where access to tertiary medical care and safe blood banks is limited.

## Conclusion

A patient with coagulation disorder is more likely to be at risk of recurrent life threatening gastrointestinal bleeding from portal hypertension and blood-borne viral diseases and are consequently more likely to benefit from an effective portal decompressive procedure. Our experience with this case suggests that this surgical procedure can be safely undertaken in clotting factor deficient patients with non cirrhotic portal hypertension if meticulous surgical hemostasis is achieved at operation and the deficient factor is adequately replaced in the perioperative period until wound healing has sufficiently progressed to prevent further bleeding.

## Abbreviations

APTT: activated partial thromboplastin time

EHPVO: extra-hepatic portal venous obstruction

EST: endoscopic sclerotherapy

FFP: fresh frozen plasma

PLRS: proximal lieno-renal shunt

PT: prothrombin time

TEG: thromboelastogram

UGI: upper gastrointestinal

## Competing interests

The author(s) declare that they have no competing interests.

## Authors' contributions

**SPC **collated the information, searched literature and wrote and revised the manuscript. **SP **assisted in literature search, writing of the manuscript, editing the final script, revising and doing the final submission. **SG, RK, PS **and **TKC **assisted in providing a critical appraisal and review of the manuscript. **RK **provided the main hematologic input.

## Pre-publication history

The pre-publication history for this paper can be accessed here:



## Figures and Tables

**Table 1:** Results of laboratory investigations in the patient with factor V deficiency and EHPVO

**Coagulation parameters before transfusion of fresh frozen plasma**	
Prothrombin time	24" (12")
APTT	54.8" (30")
**Pre-transfusion Thromboelastographic parameters**	
Reaction time (R)	24" (19–28")
Coagulation time (K)	23.5" (8–13")
Maximum Amplitude (MA)	29.5 mm (48–60 mm)
Alpha Angle	22.5 degrees (29–43 degrees)
TEG index	-3.4 (-2 to +2)
**Coagulation parameters after transfusion of fresh frozen plasma**	
Prothrombin time	15.5" (12")
APTT	33.7" (30")
**Post-transfusion Thromboelastographic parameters**	
Reaction time (R)	25" (19–28")
Coagulation time (K)	19" (8–13")
Maximum Amplitude (MA)	36 mm (48–60 mm)
Alpha Angle	26.5 degrees (29–43 degrees)
TEG index	-2.6 (-2 to +2)

APTT, activated partial thromboplastin time; EHPVO, extra-hepatic portal venous obstruction; TEG, thromboelastogram; figures in parentheses indicate control values.
